# A Novel Frameshift Variant in *SPAG1* Likely Causes Primary Ciliary Dyskinesia in Cocker Spaniels in Australia

**DOI:** 10.1002/age.70183

**Published:** 2026-08-10

**Authors:** S. E. Mead, L. E. Hambrook, R. Suryadinata, P. Robinson, C. M. Wade

**Affiliations:** ^1^ Faculty of Science, Sydney School of Veterinary Science The University of Sydney Sydney New South Wales Australia; ^2^ Advanced Vet Care Kensington Victoria Australia; ^3^ Victorian Diagnostic Service for PCD, Department of Respiratory & Sleep Medicine The Royal Children's Hospital Melbourne Parkville Victoria Australia; ^4^ Department of Paediatrics The University of Melbourne Parkville Victoria Australia; ^5^ Faculty of Science, School of Life and Environmental Sciences Camperdown The University of Sydney Sydney New South Wales Australia

## Abstract

Primary ciliary dyskinesia (PCD) is a clinical syndrome that in dogs primarily manifests as chronic respiratory disease associated with cilial malfunction. The current study employs whole‐genome sequencing and a candidate gene approach to uncover the genetic basis of PCD in three Cocker Spaniel siblings following diagnosis of their respiratory cilia by scanning electron microscopy and high‐speed video microscopy. Absence of the disorder in the parents suggested autosomal recessive inheritance. A 29 bp frameshift insertion in the eleventh exon of the candidate gene sperm‐associated antigen 1 (*SPAG1*) [NC_049234.1:g.2213788_2213789insGGCGGCGGCAAGCGGCCGGAGAGGGGCGC] was identified as likely causative for PCD in this family. The 29 bp frameshift variant was unobserved in a public variant call file including 1987 dogs from the Dog10K resource however an in‐frame insertion was sometimes observed at the same locus. A Cocker Spaniel with similar symptoms from a different family tested negative for the identified variant suggesting that there are multiple causes for the condition in Cocker Spaniels.

Primary ciliary dyskinesia (PCD) (OMIA:000573‐9615) is a disease affecting the motile cilia throughout the body (Bell et al. 2016). PCD is caused by defects in the structure or function of the motile cilia (Anderegg et al. 2019; Jung et al. 2023), which move substances across the surface of cells (Christen et al. 2023). Motile cilia are present in the respiratory tract, middle ear, auditory tube, reproductive tract, brain ventricles, and spinal cord (Bell et al. 2016; Anderegg et al. 2019). The predominant clinical sign associated with PCD in mammals is defective respiratory mucokinesis caused by decreased ciliary function, specifically caused by defects of the inner and/or outer dynein arms of the cilia (Bell et al. 2016; Anderegg et al. 2019; Haller and Guscetti 1994; Reichler et al. 2001). Three female Cocker Spaniel siblings presented with chronic rhinitis/bronchitis from day three of life that resisted standard veterinary treatment. Pathophysiological examination of bronchial and nasal mucosal cellular samples using high‐speed video microscopy and electron microscopy conducted by the PCD diagnostic unit of the Royal Children's Hospital, Melbourne confirmed the diagnosis of PCD (Table 1, Figures S1 and S2A–D). Situs inversus was not observed in the affected pups.

**TABLE 1:** Ciliary function phenotypes of primary ciliary dyskinesia affected Cocker Spaniels sequenced in this study.

Identifier	Family	Ciliary function phenotype	Source
80439	1	Absence of both inner and outer dynein arms in most cross‐sections. Cilial beat largely immotile 4.59 Hz. No situs inversus	Electron microscopy this study
80440	1	Absence of both inner and outer dynein arms in most cross‐sections. Cilial beat absent 0 Hz. No situs inversus	Electron microscopy this study
80445	1	Absence of both inner and outer dynein arms in most cross‐sections. Cilial beat mostly absent 1.5 Hz. No situs inversus	Electron microscopy this study
80446	2	Electron microscopy confirmed structural abnormalities of cilia with an outer dynein arm defect. No situs inversus	Bell et al. (2016) (Case 1 Figures 2, 3 and Table 1)

DNA was purified and sequenced from eight individuals, including seven individual Cocker Spaniels from the extended family (Family 1) and one distantly related previously diagnosed case (Bell et al. 2016). Samples included five littermates (3 affected females, two normals: one male and one female). The dam and the paternal granddam were also sampled (Figure 1A). The litter included two further males that were reported to be clinically normal. The sire was clinically normal but unavailable for sampling. All individuals were sequenced with Illumina HiSeq whole‐genome sequencing by the Ramaciotti Centre (Sydney, Australia). Sequences were aligned to UU_Cfam_GSD_1.0 (Wang et al. 2021), and variants were called using recommended protocols with default parameters (Li and Durbin 2009; Li et al. 2009; McKenna et al. 2010). Candidate genes were identified using Online Mendelian Inheritance in Man (OMIM) Phenotypic Series PS244400 (accessed October 21 2025) (Amberger et al. 2015). Variants were located on UU_Cfam_GSD_1.0 from the longest transcript boundary for each gene with 500 kilobase padding (Table S1). Regions considering specific genes (rather than mapped loci not localized to a gene) consistent with the recessive hypothesis were considered further. Variants in the specified regions were carefully examined, identifying variants in exons, splice sites, and untranslated regions of transcripts. Sequences were specifically examined at previously reported canine PCD loci identified in OMIA (Anderegg et al. 2019; Christen et al. 2023; Hedgespeth et al. 2021; Merveille et al. 2011; Nicholas et al. 1995).

**FIGURE 1:**
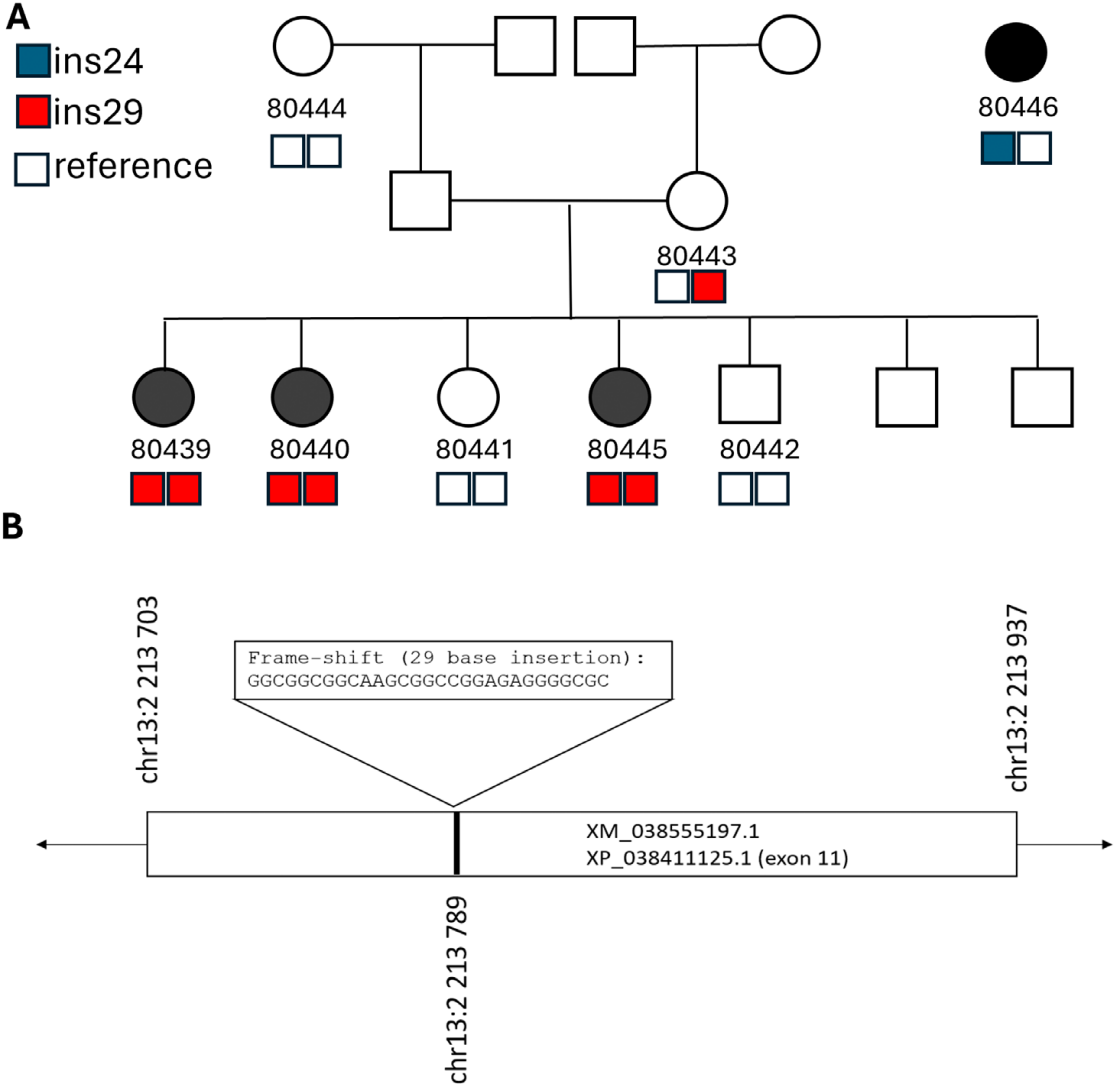
(A) Three‐generation pedigree shows a frameshift variant allele (red) segregating with clinical Primary Ciliary Dyskinesia (PCD) in a Cocker Spaniel family but not present in an unrelated case. (B) A 29 bp insertion in *SPAG1* exon 11 is likely functional for PCD in one Cocker Spaniel family. Positions are in UU_Cfam_GSD_1.0 coordinates.

All cases were homozygous for the reference allele at previously reported canine PCD variant sites *NAT10* (XM_038563052.1:c.1092A>C), *NME5* (XM_038552280.1:c.43delA), *CCDC39* (XM_038583459.1:c.286G>A), and *STK36* (XM_038585732.1:c.2868G>A). Of 12 036 identified variants across all candidate loci in the eight sequenced dogs, 238 variants on one chromosome (chr 13) harbouring two candidate genes (*SPAG1* and *LRRC6*) segregated in a manner consistent with the hypothesis of recessive inheritance affecting Family 1 (homozygous for a rare allele in cases, heterozygous in dam, heterozygous or homozygous for the reference allele in controls). The distantly related case (Family 2) was not homozygous alternate at either of the two chr 13 gene locations. Scrutiny of the diagnostic reports for all cases revealed a difference in phenotype between individuals of Family 1 (exhibiting defects of both inner and outer dynein arms) and that of the less related case individual (80446) (defects in outer dynein arms only) (Table 1, Figure S1).

Mutation screening identified a frameshift variant (ins29) in the gene *SPAG1* [NC_049234.1:g.2213788_2213789insGGCGGCGGCAAGCGGCCGGAGAGGGGCGC; XM_038555197.1:c.1188_1189insGGCGGCGGCAAGCGGCCGGAGAGGGGCGC] (Figure 1B, Figures S3 and S4) that segregated in accordance with recessive inheritance in Family 1. Among the tested controls, the dam was heterozygous for the frameshift, the paternal granddam was homozygous for the reference allele, and the two unaffected siblings were homozygous for the reference allele. Unrelated case 80 446 was heterozygous for the reference allele and an in‐frame insertion of 24 bp (ins24) at the same site [XM_038555197.1:c.1188_1189insGGCGAGGGCGGCGGCGGCGGCGGC]. The ins29 variant was unrecorded in a large public vcf of 1987 dogs (Dog10K) (Meadows et al. 2023). The ins24 variant was present in the Dog10K resource with an allele frequency of 0.109.

Two potential functional variants were observed in *LRRC6* (rs851898021 and rs851036964) that segregated with risk in all cases. Cases in Family 1 were homozygous for the alternate allele of rs851036964, and the 80 446 was a compound heterozygote for the variants (in *trans* by phase). The alternate variants code for missense mutations in some gene transcripts annotated by the Uppsala University GSD1.0 gene annotations (e.g., *LRRC6.3:p.P29L* and *LRRC6.3*:p.V20L, respectively) but in others are in the 5′UTR. PCD is a rare recessive disorder that is fully penetrant in homozygous or compound heterozygous mutants. As the affected genotypes at *LRRC6* were common in the Dog10K resource the variants were excluded as causal.

In humans, biallelic loss‐of‐function mutations in *SPAG1* result in defects of both inner and outer dynein arms of cilia, reproductive deficits and sometimes situs inversus (Knowles et al. 2013). The observed frameshift (ins29) mutation XP_038411125.1:p.396 is expected to result in a nonsense transcript from aa396 with significant truncation of the resulting protein occurring approximately 64 amino acids later. The normal protein has a length of 930 amino acids. Absence of the risk allele in another identified case suggests that further functional variants exist that are associated with the condition in Cocker Spaniels.

## Author Contributions

**L. E. Hambrook:** conceptualization, supervision, writing – review and editing, funding acquisition. **S. E. Mead:** investigation, writing – original draft. **P. Robinson:** investigation, writing – review and editing, formal analysis. **R. Suryadinata:** investigation, writing – review and editing, formal analysis. **C. M. Wade:** conceptualization, investigation, funding acquisition, writing – review and editing, project administration.

## Funding

This work was supported by Canine Research Foundation.

## Ethics Statement

University of Sydney Animal Ethics Committee (Approval number: 2021/2005).

## Conflicts of Interest

The authors declare no conflicts of interest.

## Supporting information


**Figure S1:** (A) Normal cilia structure and scanning electron micrographs of affected Cocker spaniel individuals (B) 80 439, (C) 80 440, and (D) 80 445.
**Figure S2:** (A) Normal ciliary beating and high‐speed video microscopy of affected Cocker spaniel individuals (B) 80 439, (C) 80 440, and (D) 80 445.
**Figure S3:** Detailed Integrative Genomics Viewer visualisation of mutation region (A) View showing 8 individuals revealing increased depth over bases chr13:2213789‐2 213 818 resulting from a 29 bp insertion in affected individuals from Family 1 that closely duplicates those bases (B) View with insertion expanded revealing agreement on the 29 bp insertion in affected individuals. Reads that show as an insertion, rather than as duplication, are the few reads that contain small sequencing errors. The true insertion sequence (part B) is a duplicate of the next 29 bp (GGCGGCGGCAAGCGGCCGGAGAGGGGCGC).
**Figure S4:** Manual alignment of sequencing reads from individual 80 439 demonstrating the presence of consistent alignment for consensus homozygosity of a 29 bp insertion sequence that is a duplication of the sequence following (blue shading). The genomic region including the sequencing reads is anchored by red shaded sequences that are unique within the genome. While the region is GC rich, the duplication is clearly revealed in manual alignment to a reference including the inserted sequence.
**Table S1:** Primary ciliary dyskinesia candidate gene regions assessed for variant calls in sequenced Cocker Spaniels in this study.
**Table S2:** Genotypes at possible functional mutation loci in Dog10K variant call file and eight locally sequenced Cocker Spaniel samples.

**Data S1:** age70183‐sup‐0002‐VideoS1.mp4.

**Data S2:** age70183‐sup‐0003‐VideoS2.mp4.

**Data S3:** age70183‐sup‐0004‐VideoS3.mp4.

**Data S4:** age70183‐sup‐0005‐VideoS4.mp4.

## Data Availability

Genome sequences can be found in the European Nucleotide Archive (PRJEB113647). Ciliary high‐speed video microscopy images are available via Figshare (https://figshare.com/s/271a2fe7b4ada5b6252f?file=65847432).
